# Neoadjuvant CD40 Agonism Remodels the Tumor Immune Microenvironment in Locally Advanced Esophageal/Gastroesophageal Junction Cancer

**DOI:** 10.1158/2767-9764.CRC-23-0550

**Published:** 2024-01-25

**Authors:** Maira Soto, Erin L. Filbert, Hai Yang, Stephanie Starzinski, Alec Starzinski, Marissa Gin, Brandon Chen, Phi Le, Tony Li, Brandon Bol, Alexander Cheung, Li Zhang, Frank J. Hsu, Andrew Ko, Lawrence Fong, Bridget P. Keenan

**Affiliations:** 1 Pyxis Oncology, Inc., Boston, Massachusetts.; 2 Apexigen America, Inc, San Carlos, California (now a fully owned subsidiary of Pyxis Oncology, Inc.).; 3 Cancer Immunotherapy Program, University of California, San Francisco, California.; 4 Helen Diller Family Comprehensive Cancer Center, University of California, San Francisco, San Francisco, California.; 5 Department of Epidemiology and Biostatistics, University of California, San Francisco, California.; 6 Division of Hematology/Oncology, University of California, San Francisco, California.

## Abstract

Sotigalimab is an agonistic anti-CD40 mAb that can modulate antitumor immune responses. In a phase II clinical trial of sotigalimab combined with neoadjuvant chemoradiation (CRT) in locally advanced esophageal/gastroesophageal junction (E/GEJ) cancer with the primary outcome of efficacy as measured by pathologic complete response (pCR) rate, the combination induced pCR in 38% of treated patients. We investigated the mechanism of action of sotigalimab in samples obtained from this clinical trial. Tumor biopsies and peripheral blood samples were collected at baseline, following an initial dose of sotigalimab, and at the time of surgery after CRT completion from six patients. High dimensional single-cell techniques were used, including combined single-cell RNA-sequencing and proteomics (CITEseq) and multiplexed ion beam imaging, to analyze immune responses. Sotigalimab dramatically remodeled the immune compartment in the periphery and within the tumor microenvironment (TME), increasing expression of molecules related to antigen processing and presentation and altering metabolic pathways in myeloid cells. Concomitant with these changes in myeloid cells, sotigalimab treatment primed new T cell clonotypes and increased the density and activation of T cells with enhanced cytotoxic function. Sotigalimab treatment also induced a decrease in the frequency of Tregs in the TME. These findings indicate that a single dose of sotigalimab leads to enhanced antigen presentation that can activate T cells and induce new T cell clones. This restructuring of the TME provides elements which are critical to the development of effective antitumor immune responses and improved clinical outcomes.

## Introduction

While immune checkpoint inhibition has resulted in improved survival across numerous cancers, many tumors possess preexisting or inducible resistance mechanisms to this approach (1). mAbs designed as agonists to immunostimulatory molecules are being developed to enhance antitumor immune responses. One target for this approach is CD40, a receptor expressed on the surface of antigen-presenting cells (APC), including both innate myeloid cells and adaptive B cells (2). In mice, CD40 agonism can mediate tumor clearance via stromal remodeling, induction of tumoricidal macrophages, and activation of CD4^+^ and CD8^+^ T cells (3, 4). Treatment with CD40 agonist antibodies induces activation of circulating CD4^+^ and CD8^+^ T cells, upregulates costimulatory molecules on B cells, and is associated with a transient reduction of circulating naïve B cells in patients (5–7). A CD40 monoclonal agonist antibody, sotigalimab (APX005M), combined with chemotherapy or chemotherapy and nivolumab has shown clinical activity in metastatic pancreatic adenocarcinoma, with an overall response rate of 31%–33% (8). Clinical response to sotigalimab and chemotherapy in patients with pancreatic adenocarcinoma is associated with an increased pre- and on-treatment frequency of circulating CD141^+^ dendritic cells (DC) and pretreatment HLA-DR^+^CCR7^+^ B cells, findings that were not seen in patients treated with nivolumab-containing regimens, implicating APCs at baseline as being important in the mechanism of sotigalimab (8). In addition, clinical responses with sotigalimab plus chemotherapy were associated with a higher frequency of circulating PD-1^+^Tbet^+^TCF1^+^ non-naïve CD4^+^ T cells and intratumoral Th1/IFNγ signature. In patients with metastatic melanoma, intratumoral sotigalimab in combination with systemic anti–programmed cell death protein 1 (PD-1) resulted in upregulation of antigen presentation molecules, increased infiltration of effector T cells, and expansion of new T cell clones shared between injected and noninjected tumors associated with clinical benefit (9). Neoadjuvant CD40 agonism in pancreatic cancer resulted in an increased density of DCs and a T cell–enriched tumor microenvironment compared to historical controls treated with chemotherapy, radiation, or no neoadjuvant therapy (7).

Esophageal and gastroesophageal (E/GEJ) junction cancers represent a heterogeneous group of malignances with a mortality rate of 47% for localized disease (10). Standard of care for resectable tumors is trimodality therapy, consisting of combined chemotherapy and radiation followed by surgery (11). With this regimen, pathologic complete response (pCR) is achieved in approximately 20% to 50% of tumors (dependent on histology) at the time of resection and correlates with survival (11–15). In a phase II trial of sotigalimab combined with chemoradiation prior to surgical resection in locally advanced E/GEJ cancer, a favorable pCR rate compared with historical data was observed (16). We hypothesize that CD40 agonism reshapes the tumor microenvironment (TME) by activating APCs which then direct a successful T cell response. To investigate this hypothesis, we performed high-dimensional single-cell assays on blood and tumor samples from a subset of patients treated on this clinical trial and report our findings here.

## Materials and Methods

### Clinical Trial and Sample Collection Details

**FIGURE 1:**
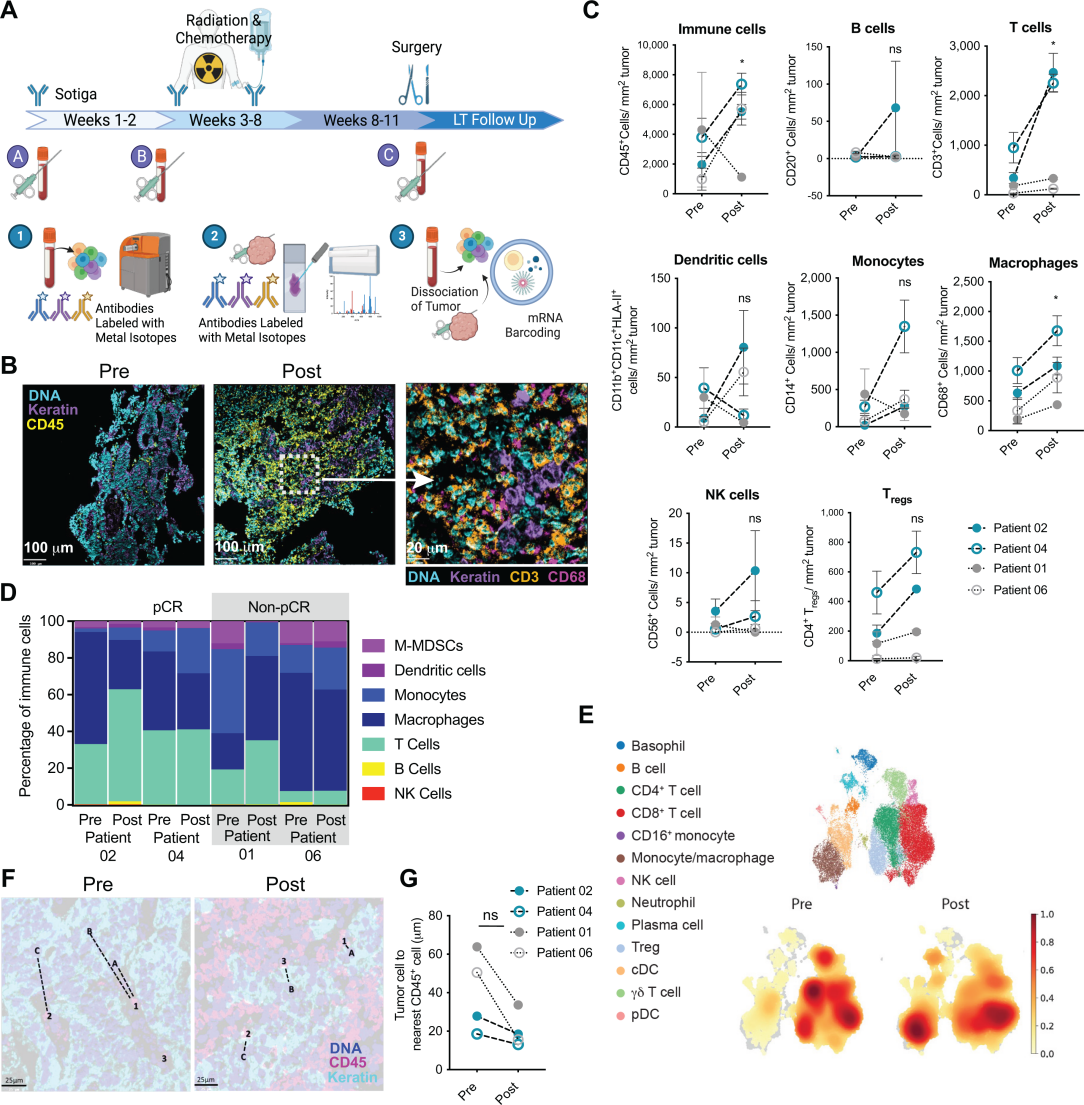
Treatment with sotigalimab increases immune infiltration into the TME, including T cells and myeloid cells. **A,** Patients were treated with sotigalimab at the initiation of the study (a), at the beginning of week 3 (b) and at week 8 (c). Chemotherapy and radiation were initiated after the second dose of sotigalimab (b). Tissue biopsies and blood samples were collected at timepoints pre-treatment, at week 3, and at surgery. Blood samples were used to assess changes to the peripheral immune system using CyTOF (1) and scRNAseq (3). Fixed tissue samples were sectioned, stained, and analyzed using MIBI (2). The tumor tissue was also dissociated into a single-cell suspension and analyzed by scRNAseq (3). **B,** Images from MIBI analysis from samples taken pre- and post-sotigalimab treatment for patient 04, showing markers for DNA, keratin (tumor cells) and CD45 for immune cells. The square at the center of the image indicates the 5x zoom image depicting DNA, keratin, CD3 and CD68 (representative image: Patient 04). **C** and **D,** The density of each immune cell type per tumor area (C) and the frequency out of total immune cells (D) is shown for each paired sample pre-sotigalimab (pre) and post-sotigalimab (post; *n* = 4). **E,** Uniform Manifold Approximation and Projection (UMAP) plot demonstrates all immune cell types within samples analyzed by scRNAseq (top). The abundance of cells within the UMAP is shown by density heat map for pre and post samples (bottom; pre *n* = 3, post *n* = 4). **F** and **G,** Distances between each tumor cell and the nearest immune cell are depicted (F) and quantified (G; *n* = 4). All statistical changes were measured by *t* test. *, *P* ≤ 0.05.

The clinical trial (NCT03165994) was initially conducted at UCSF as a single institution study under the approval of UCSF institutional review board (IRB) #17–21833 and later expanded as an industry-sponsored multisite trial. Patients, per eligibility criteria, had locally advanced, surgically resectable, (T1–3 Nx) squamous cell carcinoma, adenocarcinoma, or undifferentiated carcinoma of the esophagus or GE junction. There was a preplanned interim analysis after the first 6 patients were treated, with a plan that if 2 or more of the first 6 patients experienced unacceptable toxicities attributable to the addition of sotigalimab to CRT, then the study will be suspended and the trial design reconsidered, including but not limited to the possibility of amending the study to use a lower starting dose of sotigalimab. Treatment regimen, as diagramed in Fig. 1A was carboplatin (AUC 2)/paclitaxel (50 mg/m^2^) weekly for 5 weeks and concurrent radiation 5040 cGy in 28 fractions, and up to 4 doses of sotigalimab 0.3 mg/kg, i.v. between weeks 1–10, followed by Ivor–Lewis esophagectomy. The protocol underwent sequential amendments (for pragmatic and clinical reasons) over time, leading to adjustments in treatment administration. Written informed consent was obtained from all patients for participation in the trial and for use of blood and tumor samples in research studies, and the study was conducted in concordance with Declaration of Helsinki guidelines. Patients’ age, sex, gender, race, ethnicity, and additional tumor-related characteristics are provided in Supplementary Table S1. Blood and tumor samples were obtained from patients pre- and on-treatment (per UCSF IRB #17–21833). Biopsy samples were obtained prior to treatment initiation and at week 2 following one dose of sotigalimab; tumor samples were also obtained from surgical specimens at the time of resection. Blood samples were collected from patients at baseline, at 2 weeks following the initial dose of sotigalimab (corresponding with the biopsy), and at weeks 8–11 following chemoradiation (approximately corresponding with the surgical tissue timepoint). Because of limited tissue availability, for some experimental modalities, not all samples were available from each timepoint (see Supplementary Table S1 for details). Multiplexed ion beam imaging (MIBI) and scRNAseq were performed on tissue samples, and CyTOF and CITEseq were performed on peripheral blood mononuclear cells (PBMC).

### MIBI

Slides were stained using standardized protocols and a commercial antibody panel focused on tumor and immune cells (Ionpath Inc., Menlo Park, CA). Briefly, slides were baked at 70°C for 20 minutes, loaded onto a Histo-Tek SL Slide Stainer (Sakura), deparaffinized with xylene and then rehydrated through successive washes of reagent alcohol (100% decreasing to 70%, Sigma Aldrich) and MIBI diH_2_O (Ionpath). The slides were transferred to a PT Module (Thermo Fisher Scientific) and heated to 97°C for 40 minutes in an epitope retrieval buffer (Target Retrieval Solution, pH 9, DAKO Agilent). The slides were blocked with 5% donkey serum (Sigma Aldrich) in TBS-Tween. The antibody panel was made by diluting antibody conjugates in the blocking buffer and filtering using a centrifugal filter, 0.1 µm polyvinylidene difluoride membrane (Ultrafree-MC, Merck Millipore). The slides were stained overnight at 4°C in a humidity chamber. Slides were loaded into the slide strainer and washed three times with TBS-Tween, fixed for five minutes in 2% glutaraldehyde (Electron Microscopy Sciences), washed three times in 100 mmol/L Tris pH 8.5 (IONpath), twice in MIBI diH_2_O, and then dehydrated through a series of reagent alcohol washes (70% increasing to 100%). The stained slides were stored in a vacuum cabinet for at least 1 hour prior to loading into the MIBIscope.

### MIBI Data Analysis

Sample analysis was done on images for both pre- and posttreatment for patients 01, 02, 04, and 06; analyses that were not paired included data from pretreatment for patient 03. For each slide there was 2–3 fields of view analyzed. Images displayed in figures are all from patient 04 as a representative of the analyses done. Initial data analysis was performed with established workflows (Ionpath). Briefly, cell segmentation combines the nuclear dsDNA signal with cytoplasmic and membrane markers to accurately delineate and identify single cells in the tissue image dataset. Using a subset of image crops selected from the study, a deep learning model is trained to transform input images to response images that are smooth. The watershed segmentation algorithm is then applied on the response images to generate smooth cell boundaries. Boundaries were manually reviewed by visualizing nuclear, membrane, and cytoplasmic signals. The result is an image in which each pixel is either assigned an integer value corresponding to a unique cell instance or 0, indicating a cell is not present. Cell classification was then performed with a custom pipeline written in Python 3.7 (RRID:SCR_001658). A total of 25 cell populations were classified. The accuracy of cell classification was visually verified by Ionpath scientists with expertise in immunology and pathology.

### Mass Cytometry (CyTOF)

Cryopreserved PBMC samples were thawed, viably stained with cisplatin and fixed with paraformaldehyde (PFA). The fixed samples were barcoded with palladium isotopes, combined, stained extracellularly and intracellularly with heavy metal-labeled antibodies (Supplementary Table S2) in preparation for acquisition. On day of acquisition, the samples were washed from an excess of permeabilization buffer containing intercalator and PFA. We then acquired combined samples at 1 × 10^6^ cells/mL of calibration beads (catalog no. 201078, Fluidigm/Standard Bio Tools) in milliQ water for acquisition on a mass cytometer (Helios, Fluidigm/Standard Bio Tools). Acquired data was pre-processed using the premessa R-package (https://github.com/ParkerICI/premessa) to utilize bead-base normalization, remove beads, and debarcode samples. Following preprocessing of the data, Cytobank (RRID:SCR_014043; ref. 17) was used to manually gate cell populations, determine Boolean threshold values for functional markers and create landmark nodes for statistical SCAFFoLD analysis. We then extracted fcs files containing the live cells for each sample from Cytobank. Using the SCAFFoLD R-package (https://github.com/SpitzerLab/statisticalScaffold; ref. 18), we clustered cells from all samples into 200 unsupervised clusters. Statistical analyses were completed to determine cell cluster and protein expression frequency significances for each functional marker and transformed to log_2_-fold change between analyzed groups. The statistical data generated from the SCAFFoLD analysis was visualized into a heat map to display the cell cluster and protein expression frequency significances between the analyzed groups as described previously (19).

### Preparation of Samples for scRNAseq

Tumor tissue samples were digested in RPMI containing Collagenase I & II (0.1 mg/mL, Thermo Fisher Scientific) and DNAse I, minced, and digested for one hour using the GentleMACS system (Miltenyi Biotec). Isolation of live cells was performed using MACS LS columns (Miltenyi Biotec). Blood samples were processed using ficoll (Cytiva); after centrifugation, the PBMC layer was isolated and cryopreserved in cell media with human serum and DMSO. Previously frozen PBMCs were thawed using media containing RPMI, heat-inactivated sterile filtered human serum, penicillin–streptomycin, nonessential amino acids, sodium pyruvate, and l-glutamine (CHM media). Samples were then incubated with DNAse I (15 U/mL, Roche). After washing and counting, PBMCs were incubated in staining buffer (1 mmol/L EDTA and 2% heat-inactivated FBS in Dulbecco PBS; Thermo Fisher Scientific) with TruStain FcX (Fc Receptor Blocking Solution, BioLegend) and Total-Seq C anti-human Hashtag antibodies (Supplementary Table S3; BioLegend, see Table for RRID) for a final hashtag dilution of 2000x. A total of 1 × 10^6^ cells from 9 unique samples were combined and stained with one pooled cocktail containing a custom panel of 197 antibody–oligonucleotide conjugates targeting cell surface proteins of interest (Supplementary Table S3; BioLegend) as per manufacturer protocols. Samples from different individuals and timepoints were randomly mixed across experiments to minimize batch and confounding effects. Droplet-based single-cell RNA sequencing (scRNAseq) was performed using the 10X 5′ Reagent Kits version 1, according to manufacturer instructions. 10X 5′ kits for feature barcoding and TCR and BCR amplification were used to generate libraries per manufacturer instructions.

### Sequencing and scRNAseq Analysis

All sequencing was performed on an Illumina NovaSeq S4 sequencer with paired end 200 base pair read length and 25,000 reads per droplet for gene expression and 5,000 reads for TCR/BCR/protein libraries. CellRanger version 3.1.0 (10x Genomics, Genome Build: GRCh38 3.0.0) was used to align the raw sequencing data. For demultiplexing for PBMC scRNAseq data, we used the hashtag method using a threshold method for determining the identify of each single cell and removing doublets (20). For tumor samples, we used doubletdetection to identify doublet cells from the single cell data (21). We used the scanpy (22) data analysis pipeline for pre-processing and analysis of scRNAseq data, with the following software versions: Python 3.7.12, scanpy 1.9.1, anndata 0.8.0, leidenalg 0.8.10, scvi 0.16.0, scipy 1.7.3, numpy 1.21.6, pandas 1.3.5, statsmodels 0.13.2. We applied the following cutoffs for filtering high quality cells: <10% mitochondrial genes, >400 genes expressed per cell, and excluded platelets and red blood cells. We filtered out ribosomal and mitochondrial genes and genes detected in less than 20 cells. We then used scanpy to normalize, logarithmize, and scale the data, identify highly variable genes, and perform principal component analysis. We used scVI (23, 24) for batch correction prior to k-nearest neighbor graph construction and clustering on gene expression data with a resolution of 1.0 using the leiden algorithm. For proteins in the CITEseq dataset, we normalized and logarithmized the data. For specific comparisons of differential gene expression between cell types, we used MAST to calculate fold change and significance, based on a model incorporating cellular detection rate (based on number of genes per cell) as a covariate (25). For gene ontology analysis, we used an adjusted *P* value cutoff of <0.05 and a log fold change of 0.1 for differentially expressed genes, and the proportion of enriched genes within each pathway was calculated (26, 27). Gene scores were performed with the scanpy rank genes function and gene lists based on GO pathways M12919, M23745, and M12794, and previously published T cell effector genes (ref. 28; Supplementary Table S4).

### Immune Receptor Sequencing Analysis

Sequencing data were generated using Cellranger vdj version 3.0.2. For TCR, only the cells with both alpha and beta chain information, and for BCR, only cells with both heavy and light chain information, were included in the analysis. The network analysis was conducted at the cell level using the NAIR software (available at https://github.com/mlizhangx/Network-Analysis-for-Repertoire-Sequencing-; ref. 29) across all samples (including both time points and both tumor and PMBC samples). Non-T and non-B/plasma cells were omitted from the analysis of TCR and BCR respectively. Pairwise Levenshtein distances for the immune receptor amino acid sequences were calculated, generating a distance matrix for each chain. Connections were established exclusively between TCRs possessing identical alpha and beta chain amino acid sequences (distance = 0) and between BCRs possessing identical heavy and light chain amino acid sequences (distance = 0). The network analysis was visualized utilizing the R packages igraph (30) and ggraph (31). To compare the number of pre-existing, induced, and persistent clones for each cell type, pairwise Pearson *χ*^2^ test was performed. Bonferroni correction was used to adjust for the multiple testing (32).

### Data Availability

Data generated in this study from scRNAseq analysis are publicly available in Gene Expression Omnibus (GEO) at GSE244748. Raw data for this study using MIBI analysis were generated at IonPath Inc. Derived data supporting the findings of this study are available from the corresponding author upon request.

### Statistical Analysis

Data generated by incorporating specific statistical analysis for the correlative specimens is presented throughout the article. Legends denote the number of samples from which data was obtained; however, for some analysis, comparisons data from single cells was used. For MIBI data, a *t* test was used for comparisons between two groups and calculated using Graph Pad Prism (RRID:SCR_002798; ref. 33). For analysis where a pair-wise or other specific analysis was used to measure statistical significance, using Graph Pad Prism, the method used is denoted in the figure legend. For frequency proportions from the scRNAseq data from blood and tumors, weighted least squares was used to adjust for number of cells sequenced in each sample.

## Results

### Sotigalimab Induces Immune Infiltration Within the TME after a Single Dose

The schema for treatment in the phase II clinical trial of sotigalimab combined with CRT in the neoadjuvant setting for E/GEJ cancer (NCT03165994, *n* = 34 total, 33 received sotigalimab and were evaluable for safety) is diagrammed in Fig. 1A. Patients were eligible if they had been diagnosed with a surgically resectable E/GEJ tumor that had not previously been treated with any anticancer therapy or surgery, had an ECOG status of 0–1, and did not have other preexisting serious comorbidities or autoimmune disease. In 29 patients evaluable for the primary endpoint of efficacy, pCR was achieved in 11 tumors (38% pCR rate) including 8 of 24 adenocarcinoma (33% pCR) and 3 of 5 squamous cell carcinoma (60% pCR; ref. 16). This compares favorably with historical rates of response (19%–23% of adenocarcinoma and 42%–49% of squamous cell carcinoma; refs. 11, 13–15).

In this report, we describe the correlative data which was available from the initial six patients enrolled in the clinical trial. Following this, the protocol was revised, and it was not possible to collect additional biospecimens; detailed sample availability can be found in Supplementary Table S1. Clinical characteristics for the patients from which samples were obtained are given in Supplementary Table S1. All six patients received a dose of sotigalimab at weeks 1, 4, and 7; the first 3 patients also received a fourth dose at week 10. CRT was administered between weeks 3–8 and was followed by surgical resection. Four of these 6 patients’ tumors achieved a pCR following sotigalimab and CRT (67%) and two had partial responses (non-pCR); there were *n* = 3 each with squamous cell carcinoma and adenocarcinoma (Supplementary Table S1).

To assess the effects of treatment on immune infiltration, we used MIBI to analyze pretreatment and posttreatment tumor biopsies after a single dose of sotigalimab. We found that treatment induced a significant increase in overall immune cell density (Fig. 1B and C), particularly in tumors with pCR. Although increases in monocytes and DCs were observed in some samples, T cells and macrophages were the only immune cell subtypes that were significantly increased posttreatment. In the case of T cells, this was mainly driven by tumors with pCR (Fig. 1C). The composition of the TME was also analyzed as proportions of all immune cells present for each individual paired sample, pre- and post-sotigalimab using the MIBI data (Fig. 1D). Although the numbers within each category of pCR/non-pCR did not allow for us to formally test the association of response with TME composition, it appears that tumors with pCR generally have a more balanced T cell to myeloid cell proportion before and after treatment. The findings seen with MIBI analysis were confirmed by scRNAseq, with observed increases in the monocytes and macrophages and alterations in the frequency of T cell subsets (Fig. 1E). Despite the trend toward increased overall density of Tregs and other T cells with MIBI, sotigalimab treatment decreased the frequency of Tregs post-sotigalimab, reflecting that the proportion of different intratumoral T cell types were altered with sotigalimab (Supplementary Fig. S1A). Using MIBI, we measured the proximity of immune and tumor cells. Following sotigalimab, the distance between immune and tumor cells decreased, indicating that the immune cells are penetrating into the tumor and are not just at the tumor periphery, a change that was seen in all samples (Fig. 1F and G).

In addition to the analysis of samples following single dose of single-agent sotigalimab, we also analyzed tumors from the surgical resection following combination sotigalimab and CRT by scRNAseq. At this timepoint, there was a paucity of both immune cells and tumor cells, including a decrease in CD4^+^ T cells and Tregs, with mostly stromal and endothelial cells present, likely representing a wound healing environment and the effects of CRT (Supplementary Fig. S1B and S1C).

### Sotigalimab Recruits and Activates Myeloid Cells in the TME

**FIGURE 2:**
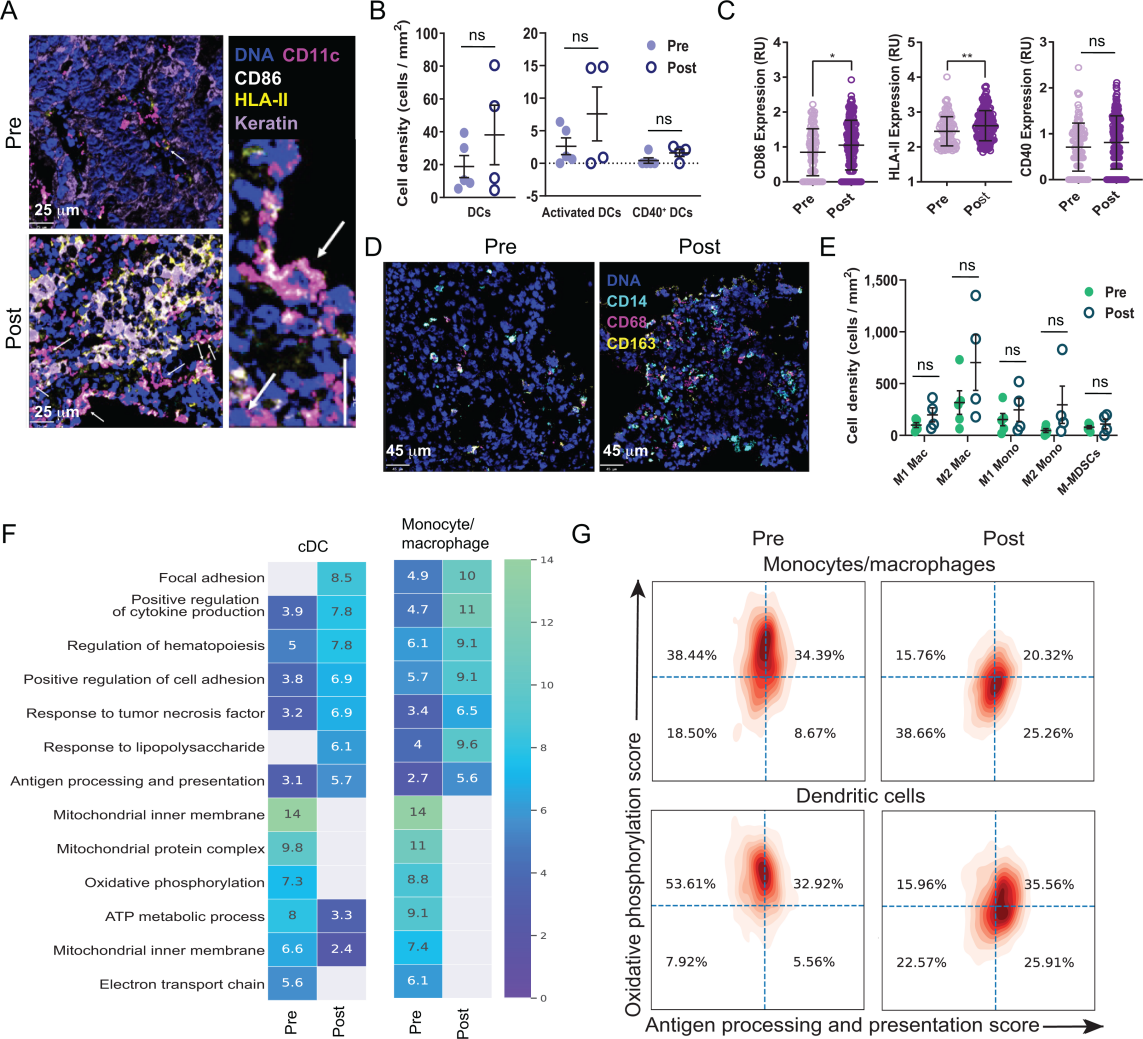
Tumor-infiltrating myeloid cells are activated post-sotigalimab with alterations in antigen processing and metabolic pathways. **A,** MIBI analysis depicts changes in cell density and upregulation of activation markers for DCs in the TME (representative image: patient 04). **B,** Quantification of DC density in the tumor pre- and post-sotigalimab by total DCs and specific subset (pre *n* = 5, post *n* = 4). **C,** Quantification of activation markers CD86, HLA-II, and CD40 expression pre- and post-sotigalimab by MIBI for all DCs pre- and post-treatment (pre *n* = 5, post *n* = 4). **D** and **E,** Visualization (D) and quantification (E) of monocyte, macrophage, and MDSC subsets using MIBI (representative image: patient 04; pre *n* = 5, post *n* = 4). **F,** The top pathways either up or downregulated post-sotigalimab in monocytes/macrophages and DCs, from scRNAseq analysis, are shown as a heat map (pre *n* = 3, post *n* = 4). The proportion of enriched genes within the pathway is shown within each box. Gray color corresponds to no enriched genes within the pathway. **G,** Individual cells were scored for genes involved in oxidative phosphorylation (*y*-axis) and genes related to antigen processing and presentation for monocytes/macrophages and DCs pre- and post-sotigalimab (*x*-axis) (pre *n* = 3, post *n* = 4). All statistical changes for MIBI data were measured by *t* test. *, *P* ≤ 0.05; **, *P* ≤ 0.01.

We further investigated the myeloid cell compartment using MIBI and scRNAseq. There was an increase in the density of total DCs, activated DCs, and CD40^+^ DCs, although this was not statistically significant likely due to small numbers of DCs overall (Fig. 2A and B). DC activation markers CD86 and HLA-II were increased post-sotigalimab, consistent with known maturation effects of CD40 agonism (Fig. 2C). CD40 expression was not significantly affected by treatment. Macrophages, monocytes, and M-MDSCs were also quantified using MIBI (Fig. 2D). Similar to DCs, there was a trend of increased cell density for each myeloid cell population (Fig. 2E). Next, we used differential gene expression and pathway analysis of tumor scRNAseq data to investigate the phenotype of infiltrating myeloid cells pre- and post-sotigalimab. Consistent with MIBI, there was a significant increase in *CD86* and HLA class II expression (Supplementary Fig. S2A). Gene expression analysis demonstrated an upregulation of other markers of mature DCs such as *CD40*, *CD80*, *CD83*, and *CD86*, post-sotigalimab. HLA class II genes, important for antigen presentation, were also among those significantly increased in the monocyte/macrophage population (Supplementary Fig. S2B). Genes related to both M1 and M2 macrophages were upregulated, as were cytokines such *CXCL8* and *IL1B*, suggesting general myeloid cell activation in the TME following sotigalimab. Pathway analysis from scRNAseq data showed that the most highly enriched pathways following sotigalimab were similar in DCs and the monocyte/macrophage populations, and included cytokine production, response to TNF, response to LPS, and cell adhesion (Fig. 2F). Pathways that were downregulated post-sotigalimab were those related to mitochondrial function and oxidative phosphorylation, the downregulation of which corresponds with myeloid cell activation and M1 polarization (34, 35). On a single-cell basis, there was a simultaneous shift to increased gene scores for antigen processing and presentation and lower gene scores for oxidative phosphorylation post-sotigalimab in both DCs and monocytes/macrophages (Fig. 2G). We also compared glycolysis and oxidative phosphorylation pathway scores on a single-cell basis and found there was an inverse shift in these pathways with treatment (Supplementary Fig. S2C). These metabolic changes are consistent with the MIBI and scRNAseq pathway analysis showing enhanced APC activation following sotigalimab treatment.

### Treatment with Sotigalimab Alters the T Cell Compartment in the TME

**FIGURE 3:**
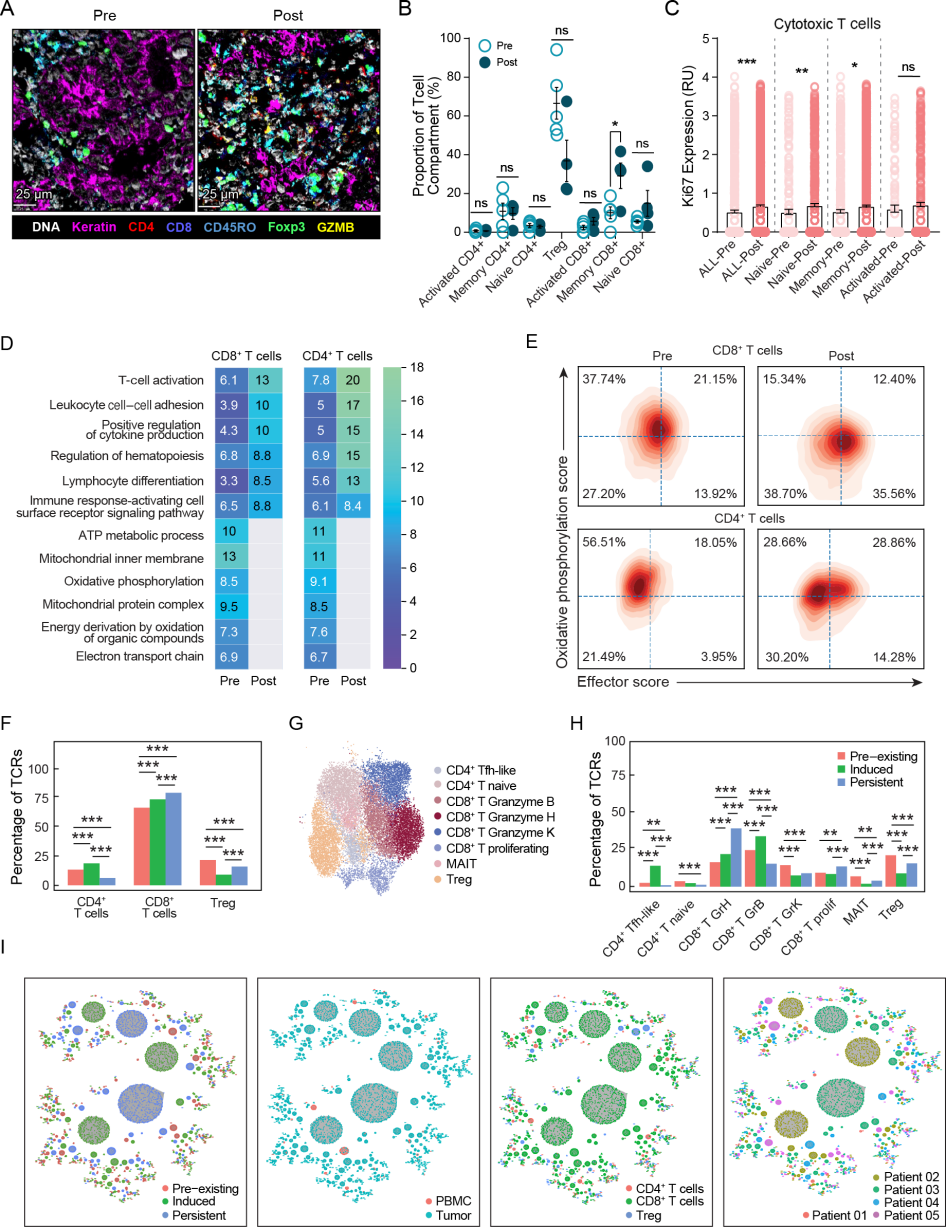
Sotigalimab induces an activated T cell infiltrate and reduces Tregs in tumors. **A,** Characterization of T cell subsets in the TME was done by MIBI analysis using markers for DNA, keratin, CD4, CD8, CD45RO, Foxp3, and Granzyme B (GZMB; representative image: patient 04). **B,** Percentage of each T cell subsets are shown for samples pre- and post-treatment, out of total intratumoral T cells, as analyzed by MIBI (pre *n* = 5, post *n* = 4). **C,** Ki67 expression in all CD8^+^ T cell subsets was quantified using MIBI analysis (pre *n* = 5, post *n* = 4). **D,** scRNAseq analysis was used to identify the top pathways either up- or downregulated in T cells (pre *n* = 3, post *n* = 4). The text inside boxes of the heat map is the proportion of genes enriched in the pathway; gray color indicates no enriched genes. **E,** Individual cells were scored for genes related to oxidative phosphorylation (*y*-axis) and T cell effector function (*x*-axis) for CD8^+^ and non-Treg CD4^+^ T cells (pre *n* = 3, post *n* = 4). **F,** Quantification of T cell clones as preexisting, newly induced, or persistent for each T cell type in the tumor (pre *n* = 3, post *n* = 4). **G** and **H,** More granular subtypes of T cells plotted in UMAP space (G) and labeled by subtype, and quantified to show distribution of preexisting, induced, or persistent clones (H). **I,** Network plot of T cell clones (paired alpha and beta chain) with cluster details (newly induced, persistent, or pre-existing clones; blood or tumor compartment; T cell type) or patient identity overlaid (tumor: pre *n* = 3, post *n* = 4; blood: pre *n* = 6, post *n* = 6). All statistical changes for MIBI data were measured by *t* test using Prism GraphPad. *, *P* ≤ 0.05; **, *P* ≤ 0.01; ***, *P* ≤ 0.001. For scRNAseq data, comparisons denoted as significant have adjusted *P* < 0.05.

Initial analysis of the TME indicated increased T cell infiltration. A deeper analysis of T cell subsets, including memory, naïve, activated, and regulatory, was performed using MIBI (Fig. 3A). Further analysis found that the ratio of the T cell subsets was also altered following sotigalimab (Fig. 3B). Before treatment, CD4^+^ Tregs were the largest subset of T cells, accounting for as high as 94% of all T cells. After treatment with sotigalimab, the proportion of Tregs decreased, which was seen in both MIBI and scRNAseq data (Supplementary Fig. S1A; Fig. 3B), and there was an increased proportion of memory CD8^+^ T cells. Increased expression of Ki67 was also seen across the CD8^+^ T cell populations, particularly in naïve and memory, suggesting that T cell proliferation could be contributing to this increase within tumors (Fig. 3C). There were no significant changes in Ki67 expression in CD4^+^ T cells. Gene expression analysis of tumor scRNAseq data demonstrated a significant upregulation in pathways related to T cell activation and differentiation, cell adhesion, and cytokine production post-sotigalimab for both CD8^+^ and CD4^+^ T cells (Fig. 3D). Specific genes upregulated in these CD8^+^ and CD4^+^ T cells post-sotigalimab included markers of activation and effector function such as *GZMB*, *IRF1, GNLY,* and *PRF1.* There was also evidence of T cell activation and potentially exhaustion as demonstrated by upregulation of *CTLA4*, *TIGIT*, and *LAG3* (Supplementary Fig. S3A). In comparison, pathways related to mitochondrial activity and oxidative phosphorylation were downregulated post-sotigalimab (Fig. 3D), an indication of T cell maturation from a naïve to effector phenotype (36, 37). In both CD8^+^ and CD4^+^ T cells, there was a simultaneous shift to decreased oxidative phosphorylation score and increased T cell effector (28) and glycolysis scores post-sotigalimab (Fig. 3E; Supplementary Fig. S3B).

Given the higher frequency of T cells in responders, in an exploratory analysis due to small sample size, we next compared alterations in T cell subsets by response status. Interestingly, in the tumors that achieved pCR, there was a trend of less Tregs at baseline, and posttreatment there was a shift to a higher proportion of CD8^+^ T cells and lower proportion of Tregs, whereas this pattern was not consistently found in the non-pCR tumors (Supplementary Fig. S3C). Further characterization of CD4^+^ and CD8^+^ T cells in the TME at baseline, by response status, was explored with the scRNAseq analysis (Supplementary Fig. S3D). In tumors that achieved pCR, both CD4^+^ and CD8^+^ T cells expressed higher levels of genes related to activation, such as *GZMB* and *TIGIT* pretreatment, and CD8^+^ T cells had lower *PDCD1* expression at baseline; however, the sample number is too small to make definitive conclusions about baseline biomarkers of response.

### Sotigalimab Treatment Induces Novel T Cell Clonotypes in the TME

Given the effects of sotigalimab on T cell phenotype, we investigated T cell specificity with single-cell TCRseq. First, we analyzed the percentage of preexisting, newly induced, or persistent (present both pre- and post-sotigalimab) clones in tumor and peripheral blood, calculating the percentage of the clones in each category for each cell type (Fig. 3F). Interestingly, we found that there were more newly induced and/or persistent CD8^+^ and CD4^+^ T cell clones than pre-existing clones in tumors following sotigalimab treatment; in contrast, there were fewer induced Tregs than preexisting or persistent Treg clones. We were intrigued by the findings for CD4^+^ and CD8^+^ T effector cells versus Tregs and investigated the changes in TCR repertoire at higher granularity. Within tumors, we found a CD4^+^ T cell state with high *CXCL13* (T follicular helper-like, “Tfh-like”) and CD8^+^ T cell subsets with increased expression of *GZMB*, *GZMH*, and *GZMK*, or proliferation-associated genes, and *TRAV1–2*-expressing MAIT cells (Fig. 3G). Applying the same analysis of TCRs to T cell subtypes, we found that there was a marked induction of Tfh-like CD4^+^ and *GZMB*^+^ CD8^+^ T cell clones and a persistence of other T cell effector clones (*GZMH*^+^ and proliferating CD8^+^ T cells) in tumors following sotigalimab (Fig. 3H). Finally, we used network analysis to analyze properties of the TCR repertoire, evaluating the cluster size (number of cells having the same TCR) in an exploratory analysis that also included TCRs recovered from PBMC (formal analysis of peripheral blood T cells was not conducted because of limited sample size). Overlay of clinical factors on the TCR network for T cells from both blood and tumor demonstrated large clusters of T cell clonotypes, particularly in the tumors, and sharing of less abundant clones between tumor and periphery (Fig. 3I). The larger clusters were induced or persistent CD8^+^ T cell clones, rather than pre-existing, suggesting induction of antigen-specific T cells within tumors.

We applied the same methods to the B cell receptor (BCR) repertoire, analyzing B cells and plasma cells from tumors. Interestingly, there was a significant increase in induced plasma cell clones and a decrease in induced B cell clones following sotigalimab treatment. No preexisting plasma or B cell clones were maintained in tumors following treatment (Supplementary Fig. S4A). Network analysis demonstrated large clusters of newly induced plasma cells in the TME post-sotigalimab and few clusters with both B cells and plasma cells (Supplementary Fig. S4B). Taken together, this suggests that sotigalimab may act by recruiting plasma cells rather than inducing maturation of B cells already residing in the TME.

### Circulating T Cells and Monocytes are Activated and Primed to Traffic to Tumors Following Sotigalimab

**FIGURE 4:**
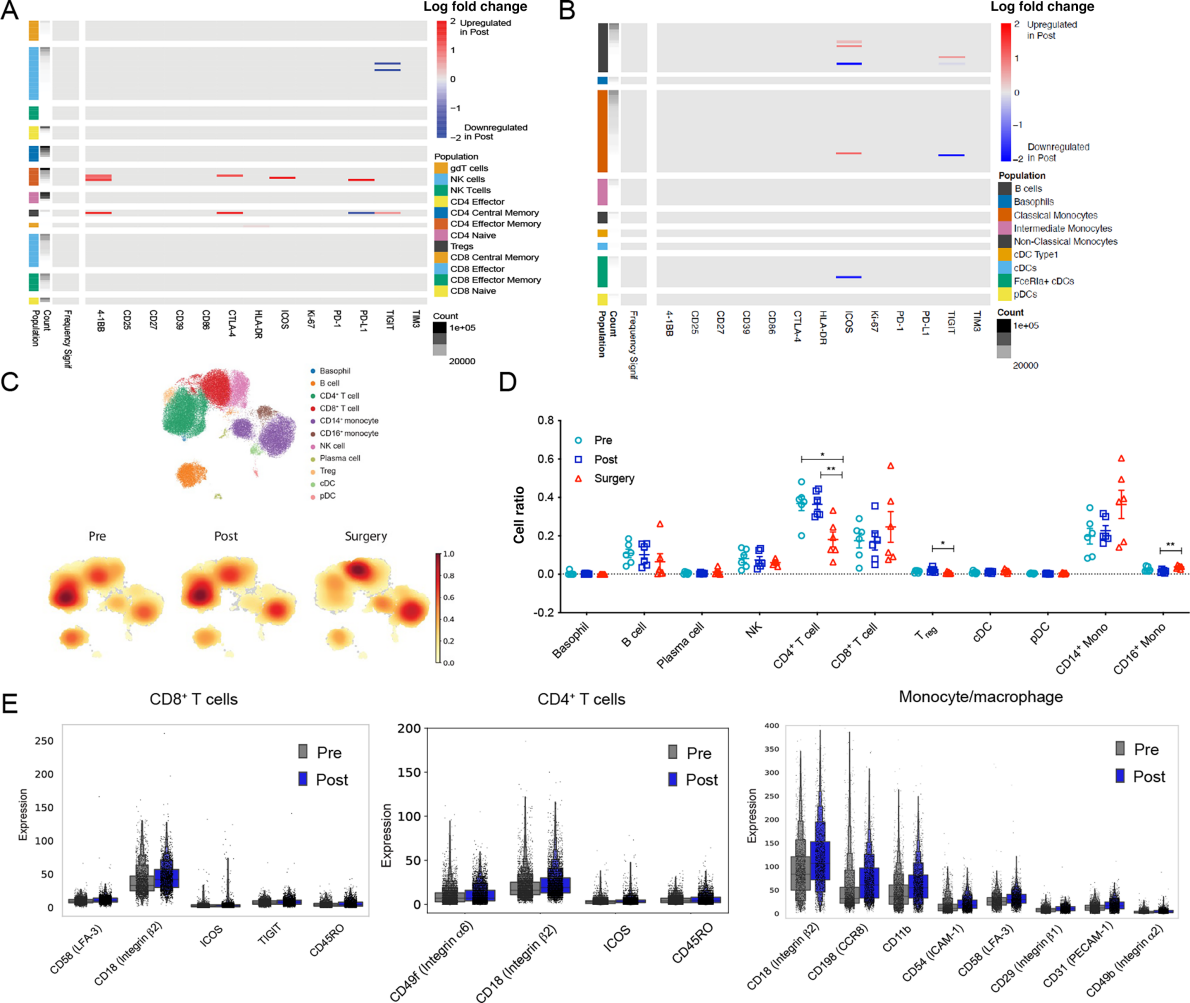
Sotigalimab induces activation and trafficking of immune cells within the blood. **A** and **B,** Heat maps of cell frequency and cell surface marker expression as analyzed by CyTOF for T and NK cells (A) and antigen-presenting cells (B; pre *n* = 6, post *n* = 6). The count column shows the number of cells in each cluster. The “frequency signif” columns indicates statistical significance for differences in cell frequency of which there were none. Red and blue in the protein columns indicate significant up- or down-regulation of expression for the indicated cell cluster. **C,** UMAP plots of PBMCs analyzed by CITEseq showing cell annotation (top) and abundance of cells by timepoint (bottom; pre *n* = 6, post *n* = 6). **D,** Quantification of cell frequency as percentage of all immune cells by timepoint from the CITEseq data (pre *n* = 6, post *n* = 6). *, *P* < 0.05; **, *P* < 0.01, unmarked comparisons are not significant. **E,** Expression of cell surface proteins indicative of cell trafficking and activation via CITEseq for CD8^+^ and CD4^+^ T cells, and adhesion molecules in CD14^+^ monocytes, for pre- and post-sotigalimab (pre *n* = 6, post *n* = 6). For scRNAseq data in E, all comparisons are significant with an adjusted *P* < 0.05.

scRNAseq with simultaneous proteomics (CITEseq) and mass cytometry (CyTOF) was used to analyze blood samples collected from patients (at baseline pre-sotigalimab, post-sotigalimab, and following sotigalimab and CRT). Evaluating frequencies of immune cell clusters identified with CyTOF (Fig. 4A and B) or CITEseq (Fig. 4C and D) did not reveal significant differences in the frequency of circulating immune cells between pre- and post-sotigalimab. There was, however, a decreased frequency of several immune cells including Tregs and CD4^+^ T cells between pre- or post-sotigalimab and surgery, and increased circulating nonclassical CD16^+^ monocytes between the post-sotigalimab and surgery timepoints, via CITEseq (Fig. 4D). As the surgical timepoint demonstrated changes consistent with the effects of chemoradiation (depletion of immune cells, particularly lymphocytes), we focused on the pre- and post-sotigalimab timepoints for further analysis of sotigalimab mechanism.

Using CyTOF, we identified increased expression of activation markers in T cells, including 4–1BB, CTLA-4, ICOS, PD-L1 expression on CD4^+^ T effector memory cells (Fig. 4A). We also observed decreased TIGIT expression on NK cells and increased 4–1BB, CTLA-4, and TIGIT and decreased PD-L1 on Tregs. CITEseq of CD8^+^ T cells demonstrated upregulation of proteins related to adhesion and trafficking (CD18, LFA-3), exhaustion and/or activation (TIGIT, ICOS), and maturation (CD45RO) post-sotigalimab (Fig. 4E). Similarly, in CD4^+^ T cells, there were increases in protein expression related to cell adhesion (integrins CD18, CD49f), maturation (CD45RO), and activation (ICOS, which was also seen via CyTOF in the CD4^+^ effector memory cluster; Fig. 4E). We did not detect enhanced measures of cytotoxicity at the RNA or protein level, suggesting that sotigalimab induces T cell activation and trafficking peripherally, and enhancement of T cell effector function occurs within the TME. In antigen-presenting cells, we detected alterations in the levels of ICOS and TIGIT on multiple populations, including DCs, monocytes, and B cells, but no significant difference in molecules related to APC function such as CD86 or HLA-DR using CyTOF (Fig. 4B). Further exploration with CITEseq revealed upregulation of adhesion molecules at the protein level including increased CD18 (integrin β2), CCR8, CD11b, CD54 (ICAM1), LFA-3, CD29 (integrin β1), CD31 (PECAM-1), and CD49b (integrin α2) (Fig. 4E).

Cell adhesion proteins were differentially expressed in baseline blood samples depending on clinical outcome, in an exploratory analysis limited by sample number. In circulating CD8^+^ T cells from patients whose tumor achieved pCR, there was higher expression of cell adhesion molecules (CD62L, CCR8, CD49d, CD18), as well as TIGIT (which was also increased in intratumoral CD8^+^ T cells in pCR versus non-pCR pre-sotigalimab; Supplementary Fig. S5A). Similarly, increased levels of adhesion molecules in circulating CD4^+^ T cells (CCR8, CD18; Supplementary Fig. S5B) and monocytes (CD18, CCR8, Cd49f, CD54, LFA-3, P-selectin, CD29; Supplementary Fig. S5C) were observed in baseline samples from patients whose tumor had pCR compared with those that did not have pCR.

### Tumor Cell Phenotype is Altered by Treatment with Sotigalimab

**FIGURE 5:**
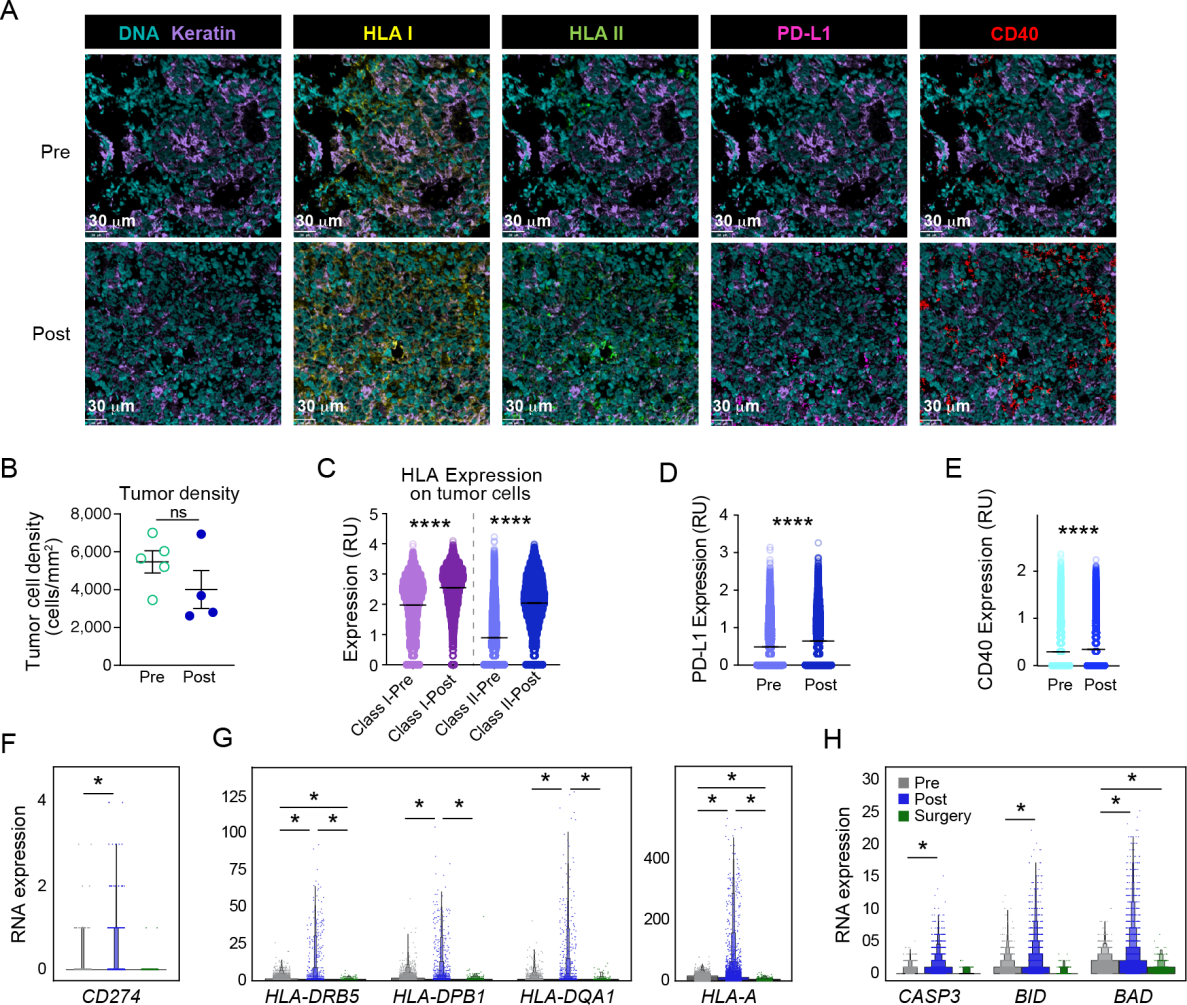
Sotigalimab results in immunomodulation and apoptosis of tumor cells. **A,** Tumor cells in the TME were characterized using MIBI analysis of keratin, HLA-I, HLA-II, PD-L1, and CD40 (representative image: patient 04). **B,** Quantification of tumor cell density by MIBI (pre *n* = 5, post *n* = 4). **C–E,** Expression of the phenotypic markers was also quantified in the MIBI analysis for HLA I and II (C), PD-L1 (D), and CD40 (E; pre *n* = 5, post *n* = 4). **F–H,** Differential expression by timepoint using scRNAseq of *CD274* (F), HLA I and II genes (G), and apoptosis genes (H) on tumor cells. For scRNAseq data, comparisons denoted as significant have adjusted *P* < 0.05, and those without an asterisk are not significant.

We next investigated the effect of sotigalimab treatment on the expression of immunomodulatory proteins by tumor cells using MIBI analysis (Fig. 5A). The immunomodulation of tumors corresponded with a decreased density of tumor cells following sotigalimab, although not significant (potentially because the image areas were selected on the basis of sufficient cells for analysis; Fig. 5B). Sotigalimab treatment induced a significant increase in both HLA class I and HLA class II proteins (Fig. 5C). PD-L1 and CD40 expression were also significantly increased in tumor cells after treatment (Fig. 5D and E). In parallel scRNAseq analysis, gene expression analysis confirmed that there was an increase in PD-L1 (*CD274*) and HLA class II genes post-sotigalimab, as observed with MIBI (Fig. 5F and G). Accordingly, we found that apoptotic molecules downstream of T cell–mediated tumor killing were increased post-sotigalimab (Fig. 5H), including *CASP3* and *BID* (38) and the proapoptotic factor *BAD* (39). In the surgical timepoint for both apoptotic and immunomodulatory genes, we found very low expression relative to pre- and post-sotigalimab (Fig. 5H), which corresponds with the lower number of tumor cells seen in pathologic specimens. The finding of tumor cell apoptosis corresponds with the increased expression of granzyme B by T cells following sotigalimab and the induction of granzyme-expressing CD8^+^ T cell clones post-sotigalimab.

## Discussion

We have demonstrated several related mechanisms by which sotigalimab may remodel the TME to a productive anticancer response. Consistent with the known mechanism of action of CD40 signaling in licensing DCs (40), antigen presentation was enhanced in the TME of E/GEJ tumors treated with sotigalimab, as demonstrated at both the transcript and protein level, in DCs, monocytes, and macrophages. Activated macrophages and DCs may subsequently induce T cell infiltration into the TME. Activated macrophages could also directly engulf and kill tumor cells (41). CD40 signaling has been shown to inhibit the induction of Tregs via interactions with DCs (42), consistent with what we demonstrated in the relative reduction of Tregs out of the total T cells in tumors. The activation of DCs by sotigalimab could both induce effector T cell responses and reduce Tregs, resulting in an increased effector to Treg ratio (43). The APC and Treg alterations corresponded with reshaping of the TME toward an activated CD4^+^ and CD8^+^ T cell environment, mainly in tumors that demonstrated pCR. Interestingly, we found the induction of new effector CD8^+^ T cell, CXCL13^+^ Tfh-like CD4^+^ T cell, and plasma cell clones in the TME post-sotigalimab, which may suggest that the formation of tertiary lymphoid structures may be a mechanism of response (44). In addition, sotigalimab administration induced changes in tumor cells, including upregulation of proteins involved in antigen presentation suggesting that sotigalimab modulates tumor cells directly or indirectly to enhance T cell–mediated killing.

Intriguingly, the intratumoral APCs and T cells had marked alterations in metabolic pathways following sotigalimab, with increased glycolysis and decreased oxidative phosphorylation, a finding not reported previously in patients treated with CD40 agonist antibodies. Naïve T cells use oxidative phosphorylation as their primary metabolic pathway and transition to glycolysis following stimulation (36, 37, 45). Activated and differentiated CD4^+^ and CD8^+^ T cells have decreased oxidative phosphorylation, whereas activated Tregs utilize oxidative phosphorylation and mitochondrial metabolism (45, 46). Furthermore, increased oxidative phosphorylation in T cells has been associated with checkpoint inhibitor resistance (47). Analogous to the T cells, metabolic pathways are used preferentially by myeloid cell subsets, with DCs and M1 macrophages using glycolysis, and MDSC and M2 macrophages relying on oxidative phosphorylation and beta-oxidation (45, 46, 48). Thus, the metabolic changes induced by CD40 agonism are consistent with induction of effector T cells and repolarization of APCs, leading to the transition of the TME from immune-suppressive to an inflammatory anticancer response.

The intratumoral findings reported here were more dramatic than changes in frequency or activation states in peripheral blood cells; however, this may be accounted for by the timing of our sampling, as the effects of CD40 agonism are immediate and transient on peripheral blood cells (5), and may not reflect the TME. However, we did demonstrate increased expression of proteins related to cell adhesion and trafficking in T cells and APCs following sotigalimab. At baseline, a higher level of these molecules on peripheral immune cells correlated with pathologic response. A notable difference between our findings and previously published data of CD40 agonism is the lack of marked changes in B cell frequency (2, 5, 49). Again, it is likely that transient B cell decreases in the blood were not captured at the later timepoint in our study. Consistent with this explanation, hematologic data from this clinical study demonstrated that there were transient decreases in circulating lymphocytes 1–2 days following sotigalimab and we demonstrated here the induction of new plasma cell clones within tumors was observed one to two weeks post-sotigalimab treatment.

The changes in APC activation and maturation observed after sotigalimab treatment are consistent with the mechanism of action of CD40 agonist antibodies and have been observed in other studies (8, 9). This study is unique, however, in that we were able to study the effects of a single dose of CD40 antibody alone in the neoadjuvant setting of E/GEJ cancer, demonstrating mechanism of action and antitumor effects in an early line of therapy. Analysis of tumor samples from a clinical trial of another CD40 agonist antibody, selicrelumab, as neoadjuvant therapy in pancreatic cancer demonstrated some notable similarities and differences with our report. While this study also found that a T cell–rich environment, mature DCs, and potential induction of new T cell clones were associated with CD40 agonism, they did not find significant differences in intratumoral Tregs, and with our use of scRNAseq, we were able to further explore gene expression programs including metabolism (7). Differences in the baseline TME of pancreatic cancer and E/GEJ cancer may account for some variability in our findings, as pancreatic adenocarcinoma has a particularly fibrotic and suppressive TME (50).

Obtaining patient samples at pivotal stages that inform the mechanisms of action of a given investigational drug can be challenging for both logistical and clinical reasons. While the total number of samples in this study was limited, we have taken advantage of the availability of a valuable set of paired patient samples and validated the results using multiple and complimentary deep immune-profiling modalities. In the clinical trial, squamous cell carcinoma histology was associated with a higher pCR rate. In our immune-profiling subset, we had an equal number of patients with adenocarcinoma and squamous cell carcinoma and an equal distribution of pathologic responses by histology. Thus, we were not powered to compare the role of tumor histology in immune response, which remains a question for future research. In addition, in this study, significant changes to the TME were observed following a single dose of sotigalimab at a set dose and our correlative analysis was limited to the samples obtained here. Future studies may examine potential effects of a higher dose or multiple dose sotigalimab.

Baseline levels of trafficking markers on peripheral blood immune cells and a favorable TME with higher effector T cell:myeloid cell ratio are potential biomarkers that can guide the development of novel therapeutics and personalize therapy for individual patients; however, our results require further investigation as they are limited by small sample size. Recently, anti–PD-1 therapy has been combined with chemoradiation in E/GEJ cancers with favorable rates of response, particularly for tumors with high PD-L1 expression (51, 52). Given that sotigalimab upregulated PD-L1 in the TME, the dual targeting of PD-1 or PD-L1 and CD40 with neoadjuvant chemoradiation in E/GEJ cancer is a rational strategy for future trials. In this study, a single dose of sotigalimab generated an immune-activated TME capable of priming and activating new immune responses, factors critical to the development of effective antitumor immune responses. These results provide evidence of the potent immune activating activity of sotigalimab and provide a rationale for CD40 agonism in combination with checkpoint inhibitor immunotherapies as a promising cancer treatment strategy.

## Supplementary Material

Supplementary Table 1 Patient clinical characteristics and sample availability.

Supplementary Table 2 Antibody details for mass cytometry.

Supplementary Table 3 Total-Seq antibody targets and barcodes.

Supplementary Table 4 Gene lists for gene scores.

Supplementary Figure 1 Supplementary Figure 1

Supplementary Figure 2 Supplementary Figure 2

Supplementary Figure 3 Supplementary Figure 3

Supplementary Figure 4 Supplementary Figure 4

Supplementary Figure 5 Supplementary Figure 5
