# Supplementary material for: Neoadjuvant CD40 Agonism Remodels the Tumor Immune Microenvironment in Locally Advanced Esophageal/Gastroesophageal Junction Cancer
Source: Cancer Res Commun. 2024 Jan 25;4(1):200–12. doi: 10.1158/2767-9764.CRC-23-0550 (PMC10809910; doi:10.1158/2767-9764.CRC-23-0550)
Supplement: Supplementary Figure 1 [file crc-23-0550-s05.pdf]

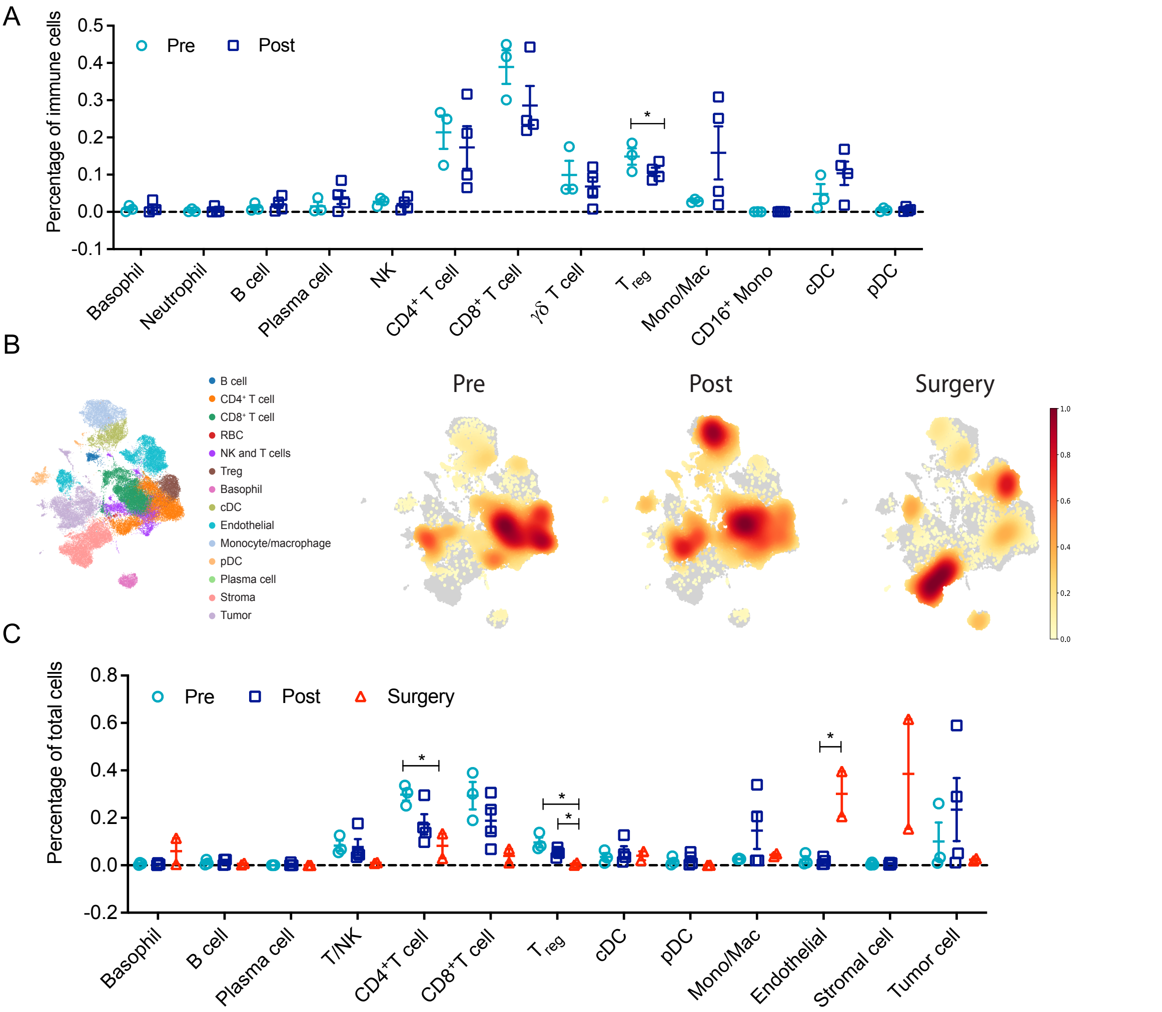

Supplementary Figure S1. Single cell RNA sequencing of pre- and post-sotigalimab biopsies and surgical samples demonstrates restructuring of the TME. A, Quantification of scRNAseq analysis for pre- and post-sotigalimab samples as the percentage of only the immune cells in tumors (pre n=3, post n=4). B, UMAP plot of all cells within the tumor, including non-immune cells (left panel). Density UMAP shows abundance of the cells by timepoint (right panels) (pre n=3, post n=4). C, Quantification of cell frequency as percentage of all cells within the tumor including non-immune cells (pre n=3, post n=4, surgery n=2). *$p \leq 0.05$, and those without an asterisk are not significant.
