# Supplementary material for: Neoadjuvant CD40 Agonism Remodels the Tumor Immune Microenvironment in Locally Advanced Esophageal/Gastroesophageal Junction Cancer
Source: Cancer Res Commun. 2024 Jan 25;4(1):200–12. doi: 10.1158/2767-9764.CRC-23-0550 (PMC10809910; doi:10.1158/2767-9764.CRC-23-0550)
Supplement: Supplementary Figure 2 [file crc-23-0550-s06.pdf]

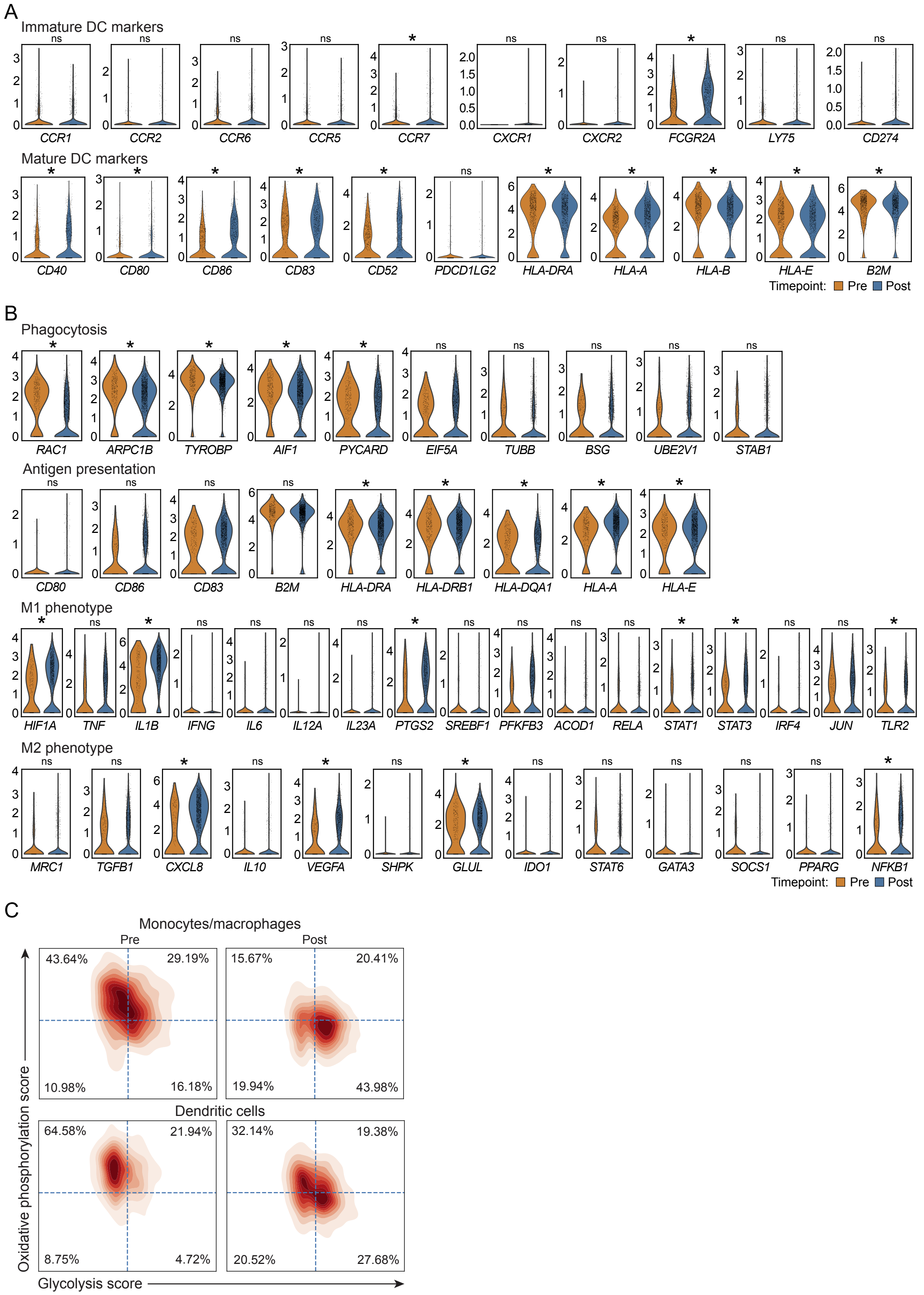


Supplemental Figure S2. Differential gene expression using scRNAseq demonstrates changes in intra-tumoral myeloid cell phenotypes. A, Differential gene expression between pre and post-sotigalimab in DCs was performed and results for select markers of immature and mature DCs are shown using violin plots (pre n=3, post n=4). B, Differential gene expression for the monocyte/ macrophage cluster between pre and post-sotigalimab was performed and expression is shown for genes related to phagocytosis, antigen processing and presentation, and M1 or M2 phenotype (pre n=3, post n=4). C, Individual cells were scored for genes related to oxidative phosphorylation (y-axis) and genes related to glycolysis (x-axis) for monocytes/ macrophages and DCs pre- and post-sotigalimab (pre n=3, post n=4). *p-value ≤ 0.05.
