# Supplementary material for: Neoadjuvant CD40 Agonism Remodels the Tumor Immune Microenvironment in Locally Advanced Esophageal/Gastroesophageal Junction Cancer
Source: Cancer Res Commun. 2024 Jan 25;4(1):200–12. doi: 10.1158/2767-9764.CRC-23-0550 (PMC10809910; doi:10.1158/2767-9764.CRC-23-0550)
Supplement: Supplementary Figure 3 [file crc-23-0550-s07.pdf]

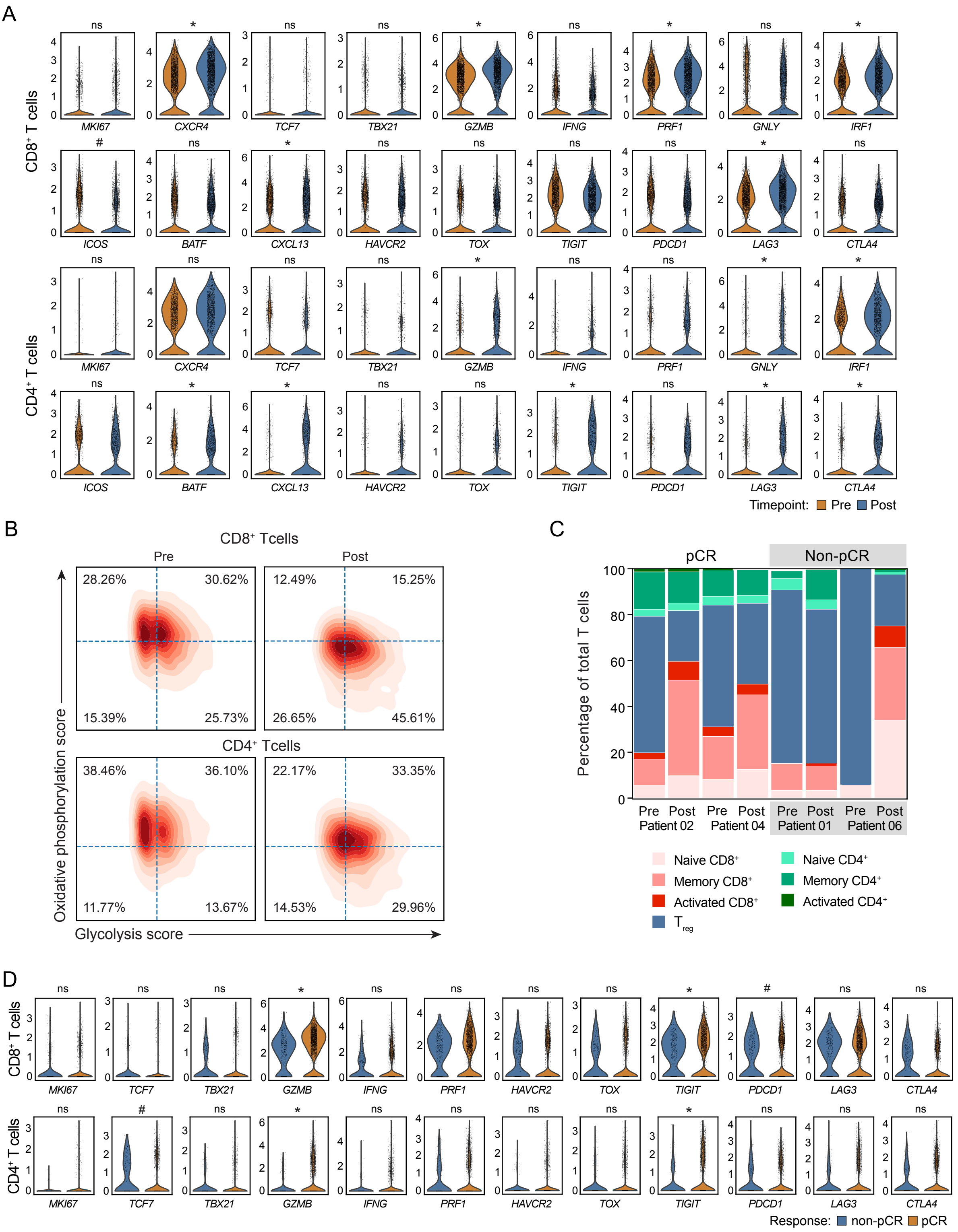

Supplemental Figure S3. Differential gene expression using scRNAseq demonstrates changes in intra-tumoral T cell phenotypes. A, Differential gene expression for CD8^{+} T cells and CD4^{+} T cells was performed for pre versus post-sotigalimab and gene expression for key markers of proliferation, exhaustion, and effector function are shown. Gene expression is plotted as normalized for each gene (pre n=3, post n=4). B, Individual cells were scored for genes related to oxidative phosphorylation (y-axis) and genes related to glycolysis (x-axis) for CD8^{+} T cells and CD4^{+} T cells pre- and post-sotigalimab (pre n=3, post n=4). C, The percentage of each subset out of total T cells is shown for individual patient samples pre- and post-sotigalimab (pre n=4, post n=4). D, scRNAseq analysis showing differential expression in specific genes involved in T cell activation in tumors that achieved pCR versus did not achieve pCR, in baseline samples (pCR=2, non-pCR=1). *p-value ≤ 0.05.
