# Supplementary material for: Neoadjuvant CD40 Agonism Remodels the Tumor Immune Microenvironment in Locally Advanced Esophageal/Gastroesophageal Junction Cancer
Source: Cancer Res Commun. 2024 Jan 25;4(1):200–12. doi: 10.1158/2767-9764.CRC-23-0550 (PMC10809910; doi:10.1158/2767-9764.CRC-23-0550)
Supplement: Supplementary Figure 4 [file crc-23-0550-s08.pdf]

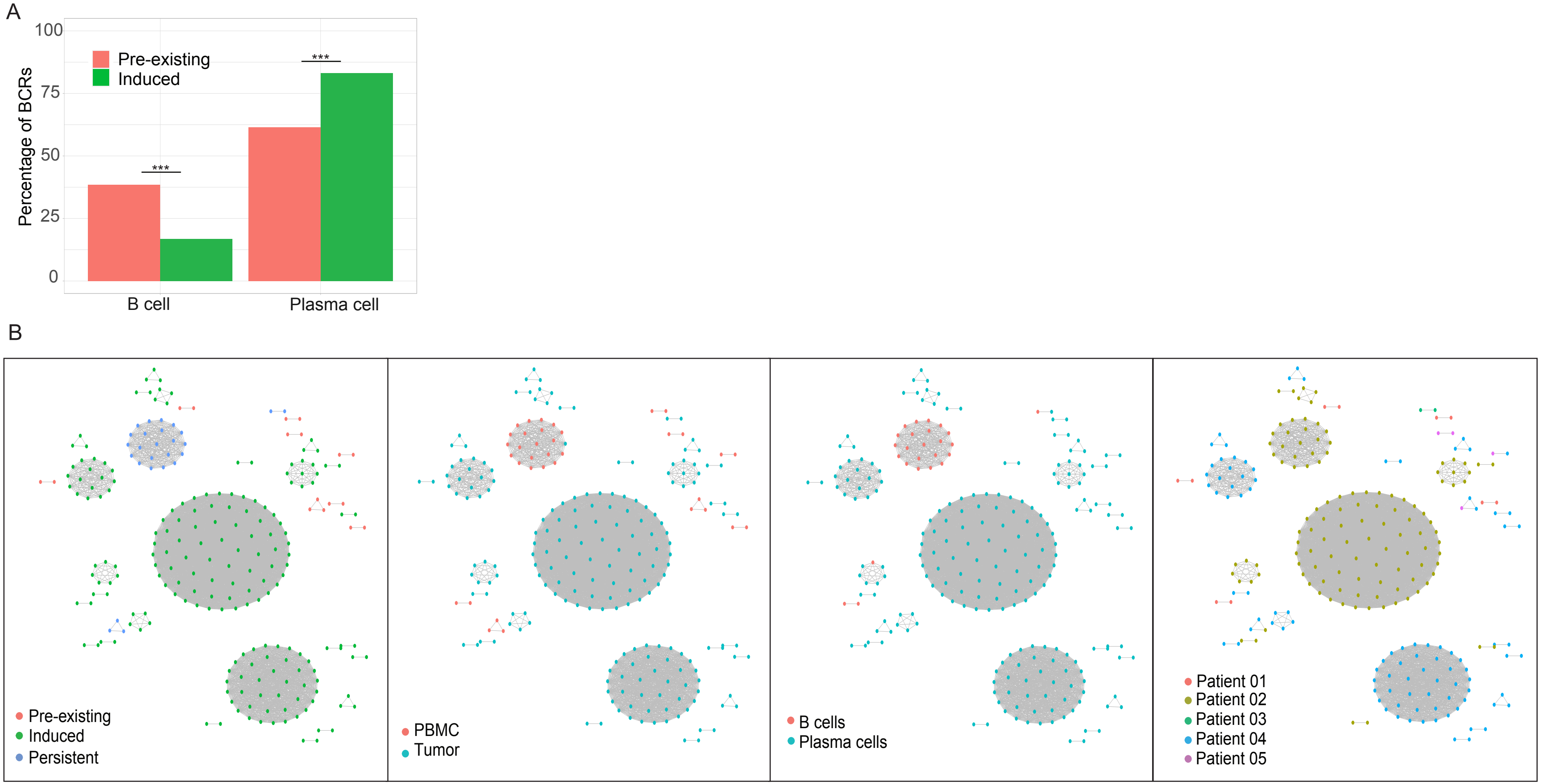

Supplemental Figure S4. Single cell immune repertoire analysis demonstrates induction of B cell clones post-sotigalimab. A, Quantification of B and plasma cell clones as pre-existing or newly induced for each B cell subtype in the tumor. There were no persistent clones detected (pre n=2, post n=3). B, Network plot of B cell clones (paired heavy and light chain) with cluster details (newly induced, persistent, or pre-existing clones; blood or tumor compartment; cell type) or patient identity overlaid (tumor: pre n=2, post n=3; blood: pre n=6, post n=6). ***adjusted p ≤ 0.001.
