# Supplementary material for: Neoadjuvant CD40 Agonism Remodels the Tumor Immune Microenvironment in Locally Advanced Esophageal/Gastroesophageal Junction Cancer
Source: Cancer Res Commun. 2024 Jan 25;4(1):200–12. doi: 10.1158/2767-9764.CRC-23-0550 (PMC10809910; doi:10.1158/2767-9764.CRC-23-0550)
Supplement: Supplementary Figure 5 [file crc-23-0550-s09.pdf]

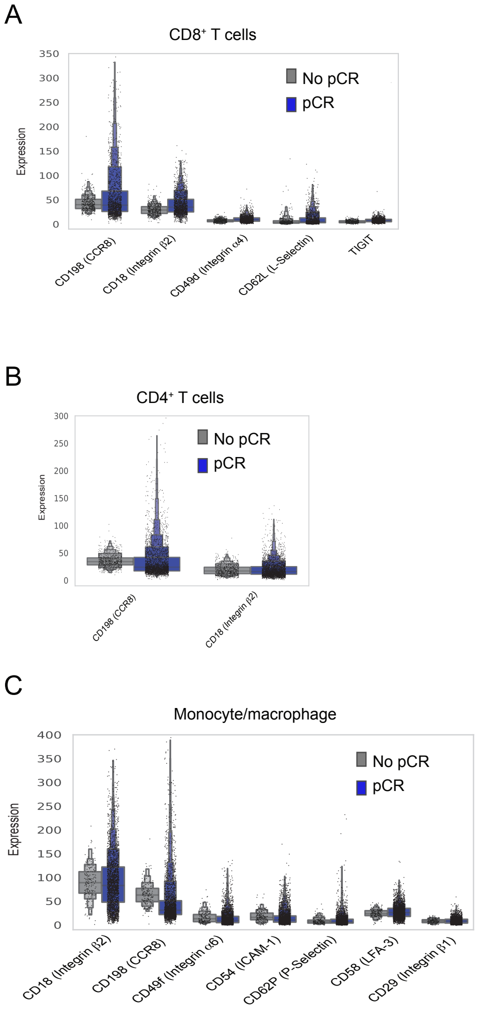

Supplemental Figure S5. Differential expression of proteins involved in cell trafficking using scRNAseq of peripheral T cell and myeloid cells by pathologic response status. A-C, Quantification of adhesion and trafficking-related proteins in circulating CD8$^+$ T cells (A), CD4$^+$ T cells (B) and monocytes/macrophages (C) based on patient response (non-pCR n=2, pCR n=4). All comparisons are significant with an adjusted p-value<0.05.
